# The Reviewer of Professor Dudley’s Essays

**Published:** 1850-02

**Authors:** 


### THE WESTERN JOURNAL.

THIRD SERIES.—VOL. V.—NO. II.

LOUISVILLE, FEBRUARY 1, 1850.

### THE REVIEWER OF PROFESSOR DUDLEY'S ESSAYS.

We have received an excellent paper on t..e surgery of gun-shot wounds, from our able correspondent, "C." It shall appear in the March No. The writer has prepared a remarkably interesting sketch of the history of military surgery, and our readers cannot fail to be pleased and instructed by it.

